# The Convention Period

**Published:** 1901-07

**Authors:** 


### Editorial.

### THE CONVENTION PERIOD.

THE passing time has brought us again to the dull season and the reassembling of the various State, interstate, and national conventions. It is somewhat paradoxical that the more inactive the period the greater the activity in dental convocations. The activity is apparently in proportion to the rise in the thermometer, and it is unfortunate that this is so, for the strain of travel and heat, after a long season of physical and mental stress, does not add either to comfort or satisfactory work.

The question is not, however, one of individual comfort, but whether these conventions are adding anything to dental progress. That they are well sustained is evident from the increasing numbers in attendance. This is the most encouraging sign, for numbers mean strength in a certain direction. That these conventions add much to the science of dentistry directly cannot be said. The immense amount of verbiage sent forth by all of them bears ample testimony to the fact that the scientific grains are not many in the bushels of chaff. This is not, or should not be, a discouraging feature, for this work is the pabulum upon which more thorough scientific labors are nourished. It is the incentive to many young men, and this is being more and more exhibited in increased mental activity, so that the time is certainly not far away when papers to be received at conventions will be required to at least have the flavor of originality in thought and a decided value through investigations. The change in this direction has been marked in the past decade, and the dental profession is beginning to reap the fruits of the long and earnest work of the dental colleges. The change in the product of all associated efforts, whether local, State, or national, is, to the mind that has lived through the various stages of development, full of astonishment, and every year is adding to this. The class of men graduating this year are unquestionably better than it was possible to produce even three years ago. While the curriculum may not have been enlarged, time has been increased, and time is an all-important factor in the development of mind.

This brings up the question, What does the National Association of Dental Faculties purpose to do this year in the matter of an increase to four years? This was alluded to in our last number, but it is essential that the members of that body should be prepared to meet this demand, which will come up in regular order of proceedings. This question has too long been deferred upon various parliamentary pretexts, and must now be met and receive careful consideration with the view, it is hoped, of final adoption. The four years' course is the only means left to relieve the difficulty now presenting at all dental colleges. The curriculum has been enlarged until further extension is impossible within the narrow limit of time now given. Dental colleges have been instituted to make dentists. They are practically making now a sort of hybrid, neither medical nor dental. This is wrong and unjust to all concerned. Give one more year, and the balance will be swerved in the right direction towards the practical. Many men are discovering this for themselves, and are voluntarily taking a four years' course, and those who have watched the result are convinced of the value of the increase of time. If the Association of Faculties acts this year, it requires no prophetic ken to see that before the first decade of the twentieth century shall have passed dentistry will have been placed on a firmer foundation than at any period in its history.

The fathers—the founders—are rapidly passing away. They have left their impress for good. The present generation of dentists are their superiors in scientific culture, but inferior in that practical ability that makes for true dental progress. The weak places can be strengthened and the new dentistry must be made superior to the old, and this can be accomplished if the remedy be intelligently grasped by those called to meet the question.

The National Dental Association has no such responsibility devolving upon it, but it has an equally important duty,—that of developing right thinking. This national body leads the associated effort of this country. It is composed of delegates from subordinate associations, and, therefore, in one sense is the nerve-centre of the entire organized body. This heavy responsibility should be met by a corresponding elevated ethical standing. Neither the old American nor the present National has been true to professional life and spirit. They have both set the bad example of bartering their proceedings for money, or its equivalent, and thus playing into the hands of the commercial side of dentistry. This has had an important influence in lowering the moral tone of the dental profession and leading it to believe that this traffic is entirely within the laws of professional ethics. Is it a cause of wonder, therefore, that so few know how to distinguish between trade and profession? The moral effect of this lay conception and practice upon dentists has been disastrous. The majority of practitioners to-day have no conception of what is meant by a true professional spirit. The two national associations mentioned are largely responsible for this, and it is time that a different example should be established. This body made a notable move last year in organizing the sections on a more scientific basis, and it is hoped this year will show marked improvement in the papers presented. Another move must be made in the interest of professional standing, and that is to print the proceedings independently of any and all journals. The time is past when these can be sold to the highest bidder. Let the Association be true to what it stands for, and the effect will be manifest in a broader life extending from national to State and from these to local organizations, and eventually to individuals, resulting in a professional spirit worthy the age and the progress made in dentistry in other directions.

While this is being written the Section on Stomatology of the American Medical Association is in session at St. Paul, Minn. This body stands for all that this article claims. There may be valid objections to it as organized, but not to its true scientific and ethical spirit. It cares nothing for number; indeed, it rather avoids the mob. Its aim is to develop the best in individuals. It does not enter the market-places and expose its work for a bid upon its proceedings. It is, in fact, true to the highest professional idea. While it may not be the best thing to imitate the methods of this body, its spirit is worthy of full acceptance; indeed, without it further progress in the right direction is impossible.

The summer will pass and, possibly, the months to follow may prove the " winter of our discontent," but there is a lingering hope that the united efforts manifest in the next two months may lay, at least, the foundation for a dentistry broad in its culture, generous in its spirit, and effective in its work.

